# Post-deployment effectiveness of malaria control interventions on *Plasmodium* infections in Madagascar: a comprehensive phase IV assessment

**DOI:** 10.1186/s12936-016-1376-5

**Published:** 2016-06-16

**Authors:** Thomas Kesteman, Milijaona Randrianarivelojosia, Patrice Piola, Christophe Rogier

**Affiliations:** Malaria Research Unit, Institut Pasteur de Madagascar, BP 1274, 101, Avaradoha, Antananarivo, Madagascar; Unité de recherche sur les maladies infectieuses et tropicales émergentes (URMITE)-UMR 6236, 27 boulevard Jean Moulin, 13385 Marseille, Cedex 05, France; Fondation Mérieux, 17 rue Bourgelat, 69002 Lyon, France; Epidemiology Unit, Institut Pasteur de Madagascar, BP 1274, 101 Avaradoha, Antananarivo, Madagascar; Institute for Biomedical Research of the French Armed Forces (IRBA), BP 73, 91223 Brétigny-Sur-Orge Cedex, France

**Keywords:** Malaria, Prevalence, Prevention and control, Cross-sectional studies, Health surveys

## Abstract

**Background:**

Because international funding for malaria control is plateauing, affected countries that receive foreign funding are expected to maintain a constant budget while continuing to reduce *Plasmodium* transmission. To investigate the appropriateness of a malaria control policy in Madagascar, the effectiveness of all currently deployed malaria control interventions (MCIs) was measured.

**Methods:**

A nationwide cross-sectional survey was conducted in 2012–2013 at 62 sites throughout Madagascar. A total of 15,746 individuals of all ages were tested for *Plasmodium* infection using rapid diagnostic tests and were interviewed about their use of long-lasting insecticidal nets (LLINs), indoor residual spraying (IRS), intermittent preventive treatment of pregnant women (IPTp), and exposure to information, education and communication (IEC) campaigns. The association between *Plasmodium* infection and MCI exposure was calculated using multivariate multilevel models, and the protective effectiveness (PE) of an intervention was defined as one minus the odds ratio of this association.

**Results:**

The individual PE of regular LLIN use was high and significant (41 %, 95 % confidence interval [CI] 23–54), whereas its community PE was not. The PE of IRS at the household level was significant in one transmission pattern only (44 %, 95 % CI 11–65), and the community PE with high IRS coverage (>75 %) was high and significant overall (78 %, 95 % CI 44–91). Using LLINs after IRS increased the PE, and the reciprocal was also true. The maternal PE of IPTp was high but non-significant (65 %, 95 % CI −32 to 91). The PE of IEC was low, non-significant and restricted to certain areas (24 %, 95 % CI −34 to 57).

**Conclusions:**

This snapshot of the effectiveness of MCIs confirms that integrated vector control is required in malaria control policies in Madagascar and suggests combining MCIs when one is questionable. Policymakers should consider the local effectiveness of all deployed MCIs through a similar phase IV assessment.

**Electronic supplementary material:**

The online version of this article (doi:10.1186/s12936-016-1376-5) contains supplementary material, which is available to authorized users.

## Background

Following a sharp increase in international funding, successful malaria control has been observed over the previous decade, with certain countries (e.g., Madagascar in 2007) approaching malaria pre-elimination [1, 2]. Because international funding for malaria control is reaching a plateau [2], malaria control programmes must improve their strategies for combating malaria in a cost-effective manner. To achieve malaria elimination, policy makers should be informed of the effectiveness of each control intervention in reducing *Plasmodium* infections within the overall population of each country. Demographic and Health Surveys (DHSs) and Malaria Indicator Surveys (MISs) partly provide such information [3, 4], although they are limited to target populations (i.e., children under 5 years of age and women of child-bearing age), whereas the entire population must be considered for the elimination of malaria parasites [5, 6]. Additionally, the effectiveness of malaria control interventions (MCIs) is usually measured separately and in geographically restricted areas despite the simultaneous deployment of MCIs. Therefore, the project described in the present manuscript aimed to evaluate the individual effectiveness of current MCIs in reducing *Plasmodium* infections through a comprehensive study. This study, termed MEDALI (for Mission d’Etude des Déterminants de l’Accès aux Méthodes de Lutte antipaludique et de leur Impact), was performed in Madagascar during 2012–2013. The design and methodology of the MEDALI project, assessment of malaria infection, morbidity and mortality, and evaluation of the coverage of MCIs have been previously described [5]. The present manuscript depicts the evaluation of the effectiveness of long-lasting insecticidal nets (LLINs), indoor residual spraying (IRS), intermittent preventive treatment of pregnant women (IPTp), and information, education and communication (IEC) campaigns against Plasmodium infection. These MCIs are key interventions in Madagascar, most of them having progressively been adopted during the 2000’s, in parallel with availability of international funding. IPTp was introduced in 2004. In 2006, the national recommendation for treatment of uncomplicated malaria switched from chloroquine to artemisinin-based combination therapy (ACT), together with the use of rapid diagnostic tests (RDT). In 2008, universal coverage with LLINs was scaled up at national level, and IRS with pyrethroids was deployed in the Central Highlands and Fringe transmission patterns. In 2009–2010, IRS was extended to some South and Western districts, while it was down-scaled to focalized IRS in the Central Highlands. In parallel with deployment of these MCIs, IEC campaigns were reinforced.

## Methods

A complete description of the methodology of MEDALI’s cross-sectional survey is published elsewhere [5]. Below are the key points.

### Study sites

**Fig. 1:**
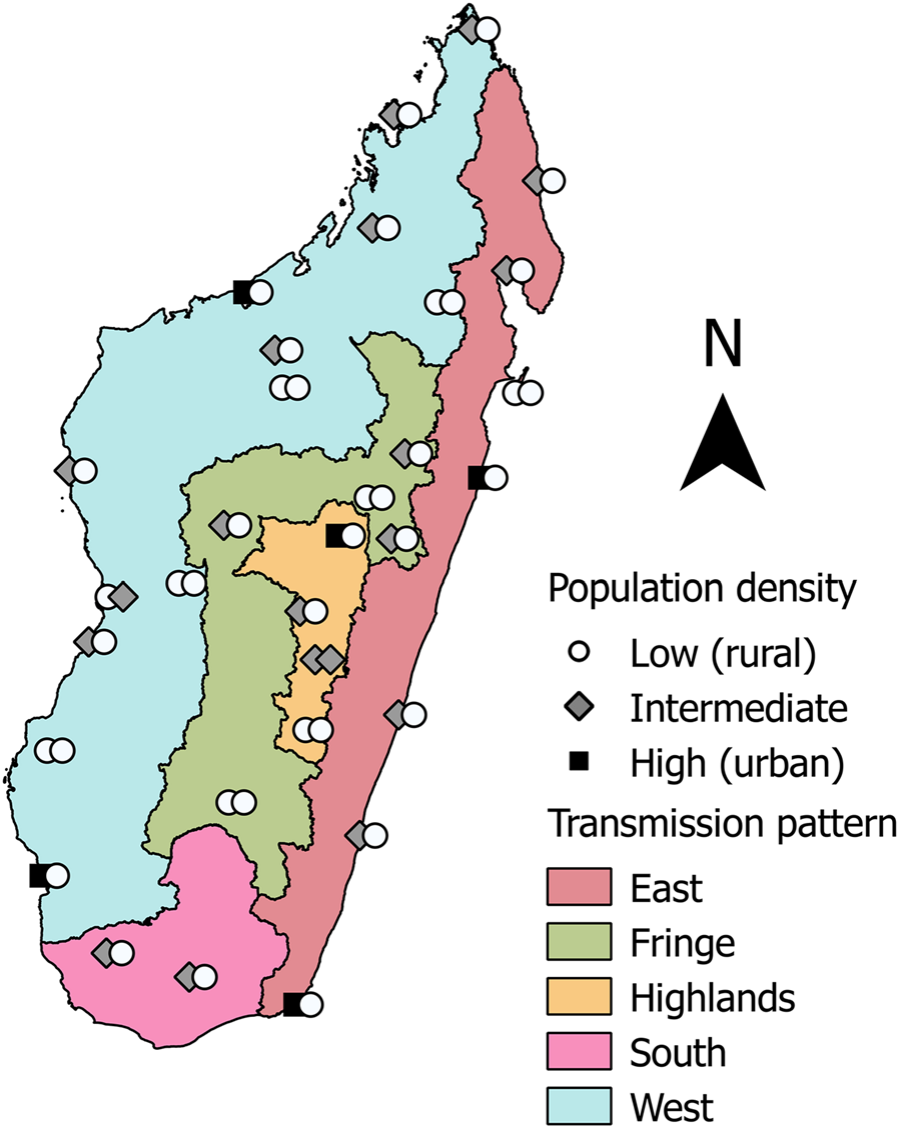
Malaria transmission patterns in the districts of Madagascar and MEDALI study sites and their population densities

Study sites were selected from a pre-existing network of sentinel health centres (SHC) for the surveillance of fever-associated diseases [7]. One SHC in each locality where at least one SHC existed was selected, and two study sites were randomly selected near each of the 31 SHC, for a total of 62 study sites [5, 8] (Fig. 1). The two coastal regions (east and west) exhibit hyperendemic malaria patterns. In the central highlands and the south, transmission patterns are unstable, episodic or epidemic. In the fringe areas at intermediate altitudes, transmission is limited to the rainy season. Coastal areas were investigated during September–October 2012, and other areas were investigated between November 2012 and January 2013.

### Sample size calculation

A total sample size of 13,950 is sufficient to detect an odds ratio (OR) of 0.7 for malaria RDT positivity with a power of 80 % according to the following parameters: baseline proportion of positive RDTs [or parasite rate (PR)] of 5 %, intervention coverage of 50 %, cluster effect of 2, and alpha risk of 5 % [9]. Under the same assumptions, this sample size is sufficient to detect ORs of 0.75 and 0.8 with powers of 69 and 49 %, respectively. To achieve a total sample size of 13,950 individuals, at least 225 people from a minimum of 50 households in each of the 62 study sites were included.

### Data collection and treatment

The inclusion criteria included the following elements: ≥6 months of age; signed informed consent; and ability to take per os treatment in the case of positive RDT. The head of household or a representative and all participants answered a questionnaire about socio-demographic features and exposure to MCIs. Bed net use was defined as “use every night during the last 3 months” [5]. Household socio-economic status (SES) quintiles were created using principal component analysis (PCA) as described previously [5, 10]. Similarly, quintiles of housing permeability to mosquitoes were created using PCA based on housing construction materials and structural holes. Categories of exposure to IEC malaria messages were calculated by PCA according to one of the following types of media and time since the previous exposure: radio, poster, mobile video unit (MVU), television, leaflet or written press article, or other media/presentation [e.g., *hiragasy* (traditional Malagasy theatre), puppets, theatre, etc.]. The complete definition of all variables is available in Additional file 1. Blood was drawn from all participants by finger or heel puncture for RDT (CareStart^®^ Malaria, Access Bio Inc., Monmouth Junction, NJ, USA). The population density of each study site was determined from the WorldPop/AfriPop database [5, 11]. The surface area of the study sites was calculated by contouring clusters with a polygon extending through GPS coordinates of external households using QGIS version 2.2.0.

### Statistical analyses

Analyses were performed for the complete dataset (IEC) or limited to populations targeted by the interventions (LLIN, IRS, or IPTp). The outcome was the result of the RDT: negative versus HRP2 and/or pLDH positivity. To explore factors associated with RDT positivity, generalized estimating equation (GEE) models were fitted by considering an exchangeable within-site correlation structure using the *gee* function of R [12]. The explanatory variables were fit into backward stepwise logistic regression models, and two variables (transmission pattern and population density) were forced in all models. All of the multivariate model fits were evaluated using binned residual plots [13, 14]. Two variables were tested as potential effect modifiers for the effectiveness of the MCIs: (i) the malaria transmission pattern and (ii) individuals <5 years old. Whenever the p value of these interactions terms were <0.05, a separate analysis was conducted for the associated area or age group. The effectiveness of LLIN and IRS was tested at the individual and cluster level, i.e., high coverage (>75 % population) versus low coverage (≤75 %). The protective effectiveness (PE) of an intervention was defined as one minus the odds ratio of the exposure to this intervention as described previously [15].

### Ethics, consent and permissions

The study followed ethical principles according to the Helsinki Declaration. Informed consent was obtained from the individuals or the parents/tutors of the children before inclusion. The protocol of the present study was approved by the National Ethics Committee of the Ministry of Public Health of Madagascar (approval #CNE 57/MSANP/CE, July 24th, 2012).

## Results

### Sampled population, malaria prevalence and intervention coverage

**Fig. 2:**
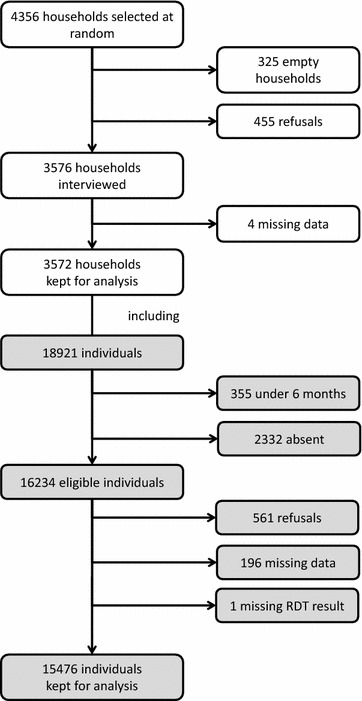
Flow diagram

The survey population included 18,921 individuals, and of the 16,234 eligible individuals, 561 (3.5 %) refused to be interviewed or sampled and 197 (1.2 %) had missing data. Therefore, the final population was 15,476 individuals (Fig. 2). Overall, a PR of 3.6 % was observed, involving *Plasmodium falciparum* in 99.6 % of cases. These results were presented and discussed in a previous publication [5]. The median surface area of the study sites was 0.4 km^2^ (interquartile range [IQR] 0.1–1.5 km^2^).

### Factors associated with *Plasmodium* infection

All of the socio-demographic variables except household permeability to mosquitoes and population density were significantly associated with *Plasmodium* infection as detected by RDT (Table 1).

**Table 1:** Bivariate and multivariate analyses of socio-demographic factors associated with RDT positivity (RDT +)

Variable/category	N	% RDT+	Bivariate	Bivariate	Multivariate	Multivariate
			Crude OR [95 % CI]	p	Adj. OR [95 % CI]	p
Age group (years)						
0–1	713	2.9	1.61 [0.84–3.09]	0.155	1.59 [0.80–3.14]	0.184
2–4	1720	3.3	1.97 [1.29–3.01]	0.002	1.94 [1.22–3.09]	0.005
5–9	2717	5.7	3.50 [2.57–4.77]	<0.001	3.57 [2.51–5.08]	<0.001
10–14	2237	5.6	3.55 [2.55–4.94]	<0.001	3.79 [2.62–5.48]	<0.001
15–19	1569	4.0	2.42 [1.76–3.34]	<0.001	2.82 [1.93–4.11]	<0.001
20–39	3699	2.4	1.42 [1.02–1.98]	<0.001	1.56 [1.08–2.25]	0.018
≥ 40	2821	1.6	1.00		1.00	
Sex						
Male	6673	4.5	1.00		1.00	
Female	8803	2.9	0.65 [0.55–0.76]	<0.001	0.66 [0.55–0.80]	<0.001
Education level						
None or unknown	2627	5.1	1.87 [1.34–2.62]	<0.001	1.95 [1.20–3.16]	0.007
Primary	6493	4.2	2.06 [1.51–2.82]	<0.001	2.01 [1.25–3.21]	0.004
Lower secondary	4302	2.6	1.48 [1.07–2.06]	0.019	1.46 [0.90–2.36]	0.124
Upper secondary/tertiary	2054	1.6	1.00		1.00	
SES quintile						
1st (poorest)	3095	6.2	1.51 [0.48–4.73]	0.483	2.49 [1.49–4.14]	<0.001
2nd	3101	4.7	1.88 [1.35–2.62]	<0.001	2.26 [1.46–3.49]	<0.001
3rd	3138	2.7	2.07 [1.51–2.83]	<0.001	1.51 [0.98–2.31]	0.061
4th	3044	2.5	1.49 [1.07–2.06]	0.018	1.65 [1.18–2.31]	0.003
5th (wealthiest)	3098	1.7	1.00		1.00	
Household permeability quintiles						
1st (most perm.)	3054	6.1	1.35 [0.91–2.01]	0.137		
2nd	3141	4.0	1.29 [0.90–1.85]	0.167		
3rd	3000	3.5	1.30 [0.96–1.76]	0.096		
4th	3032	1.8	0.85 [0.60–1.21]	0.367		
5th (least perm.)	3249	2.4	1.00			
Population density						
Low (rural)	9729	4.3	1.17 [0.50–2.77]	0.717	1.18 [0.43–3.22]	0.745
Medium	4577	2.2	1.03 [0.41–2.62]	0.948	1.03 [0.38–2.77]	0.961
High (urban)	1170	2.7	1.00		1.00	
Transmission pattern						
East	3451	4.6	7.28 [2.13–24.85]	0.002	11.29 [3.84–33.20]	<0.001
West	6654	4.7	7.16 [2.28–22.56]	<0.001	9.32 [3.49–24.93]	<0.001
South	957	3.0	4.49 [0.82–24.49]	0.083	4.20 [0.73–24.27]	0.109
Highlands	1984	1.5	2.23 [0.65–7.63]	0.203	0.98 [0.18–5.39]	0.985
Fringe	2430	0.7	1.00		1.00	

### Bed nets

The median age of LLIN was 24 months (IQR 12–36 months) and the median age of non-impregnated bed nets (NIBN) was 37 months (IQR 24–72). Of the 54 sites in areas targeted for mass distribution of LLINs, the PR was 3.9 %, and use of LLINs every night produced a significant 35 % PE (95 % CI 14–51). Significant interactions were not observed between the variables >5 years of age and LLIN use; however, significant interactions were observed between the southern transmission pattern and LLIN use (p = 0.035), and separate analyses were conducted. In the south, LLIN use was associated with a significantly increased risk of infection (OR 4.45, 95 % CI 2.10–9.40; Additional file 2). In all other areas targeted by LLIN distribution campaigns, the nightly use of LLINs produced a significant PE of 41 % (95 % CI 23–54), and the use of NIBN yielded a non-significant PE of 24 % (95 % CI −5 to 45; Table 2; Additional file 2). In five of 54 sites, LLIN coverage reached 75 %; however, this high coverage was not associated with additional community protection.

**Table 2:** Association between RDT positivity and MCI in bi- and multivariate analyses

MCI	Area	Variable	Category	N	% RDT+	Bivariate		Multivariate	
						Crude OR [95 % CI]	p	Adj.* OR [95 % CI]	p
LLIN	All targeted zones except the south	Nightly bed net use	LLIN	6780	3.7	0.55 [0.45–0.67]	<0.001	0.59 [0.46–0.77]	<0.001
			NIBN	1364	3.7	0.72 [0.53–0.98]	0.037	0.76 [0.55–1.05]	0.090
			None	4391	4.4	1.00		1.00	
		LLIN coverage	≤75 %	11,327	3.7	1.00		1.00	
			>75 %	1208	6.2	1.78 [0.50–6.34]	0.371	1.82 [0.51–6.49]	0.358
IRS	Southern transmission pattern	IRS the previous year	No	303	4.3	1.00		1.00	
			Yes	654	2.4	0.56 [0.35–0.87]	0.010	0.56 [0.35–0.89]	0.014
		IRS coverage	≤75 %	708	3.7	1.00		1.00	
			>75 %	249	1.2	0.33 [0.08–1.42]	0.136	0.14 [0.03–0.61]	0.008
	All targeted zones except the south	IRS the previous year	No	1736	1.1	1.00		1.00	
			Yes	3460	1.4	1.13 [0.74–1.72]	0.564	1.11 [0.69–1.78]	0.669
		IRS coverage	≤75 %	3662	1.6	1.00		1.00	
			>75 %	1534	0.6	0.36 [0.12–1.12]	0.077	0.20 [0.07–0.56]	0.002
LLIN and IRS	All targeted zones except the south	Nightly bed net use and/or IRS coverage	LLIN use and IRS coverage >75 %	427	0.5	0.22 [0.04–1.23]	0.085	0.14 [0.03–0.61]	0.009
			No LLIN use and IRS coverage >75 %	596	0.5	0.42 [0.10–1.82]	0.248	0.24 [0.07–0.83]	0.024
			LLIN use and IRS coverage ≤75 %	1104	1.3	0.78 [0.44–1.37]	0.385	0.93 [0.58–1.49]	0.764
			NIBN use and IRS coverage ≤75 %	160	1.9	0.96 [0.08–11.71]	0.974	0.98 [0.09–10.67]	0.985
			No bed net use and IRS coverage ≤75 %	1650	1.4	1.00		1.00	
IPTp	All targeted zones	≥1 dose IPTp	Yes	103	1.9	0.48 [0.09–2.42]	0.370	0.34 [0.05–2.10]	0.244
			No	104	3.8	1.00		1.00	
IEC	Fringe and east	Exposure to malaria IEC	Low	2682	4.0	1.00		1.00	
			High	1779	2.6	0.70 [0.50–0.99]	0.042	0.79 [0.55–1.16]	0.230
			Very high	1413	1.7	0.65 [0.42–1.00]	0.050	0.76 [0.43–1.34]	0.345
	Whole country except the fringe and east	Exposure to malaria IEC	Low	4623	3.8	1.00		1.00	
			High	2497	4.0	1.08 [0.85–1.36]	0.543	1.08 [0.84–1.38]	0.544
			Very high	2432	3.5	0.98 [0.67–1.44]	0.916	1.09 [0.71–1.68]	0.695

* OR adjusted for age, gender, education, SES quintile, population density, and transmission pattern

### Indoor residual spraying

In the 25 sites targeted by IRS campaigns (highlands, fringe and south), the PR was 1.6 %, and membership in a household with IRS afforded a small and non-significant PE of 11 % (95 % CI −25 to 37). IRS coverage was greater than 75 in 28 % (7/25) of the sites, including one in the south. This high coverage yielded a significant community PE of 78 % (95 % CI 44–81). No significant interaction was observed between the variables <5 years old and IRS coverage; however, significant interactions were observed for the southern transmission pattern (p = 0.014). In the south, sleeping in a house that had received IRS during the previous year and living in a site with high IRS coverage provided significant PEs of 44 and 86 %, respectively (Table 2; Additional file 3). In all other areas targeted by IRS campaigns, IRS did not provide a PE at the household level; however, high IRS coverage provided a significant PE of 80 % at the community level (Table 2; Additional file 3).

### Concurrent exposure to IRS and LLINs

In areas targeted by LLIN distribution and IRS campaigns, the PR was 1.5 %. Based on the observed interactions between the effectiveness of LLINs and the southern transmission pattern, an analysis was conducted in a subpopulation from 16 sites, excluding the southern transmission pattern. In this group, IRS was effective at the community level and not at the household level (Table 2); therefore, the former variable was retained in the analysis. Using LLINs in a zone with high IRS coverage provided a significant PE of 86 % (95 % CI 39–97; Table 2; Additional file 4). Living in a zone with high IRS coverage but not using LLINs was still associated with a significant PE of 76 % (95 % CI 17–93). In this subset, using LLINs in a zone with low IRS coverage produced a non-significant PE of 7 % only (95 % CI −49 to 42; Table 2).

### Intermittent preventive treatment of pregnant women

In areas targeted by IPTp (i.e., all of the transmission patterns except that of the central highlands), among 207 pregnant women (after the first trimester) and women who had delivered the previous month, administration of at least one dose of IPTp provided a non-significant PE of 66 % (95 % CI −110 to 95; Table 2; Additional file 5). The PR was 2.9 % in this subpopulation. No significant interaction was observed between IPTp and transmission pattern.

### Information, education and communication campaigns

A high level of individual or caretaker exposure to malaria IEC messages provided a non-significant PE of 2 % (95 % CI −40 to 31, p > 0.1). A significant interaction was observed between the eastern and fringe transmission patterns. In these areas, a high or very high level of exposure to IEC yielded a significant 30 or 35 % PE in bivariate analysis respectively, but the significance dropped and PE decreased to 21 % (95 % CI −16 to 45) and 24 % (95 % CI −37 to 57) respectively in multivariate analysis (Table 2; Additional file 6). No PE of the IECs was observed for the other sites.

An analysis of the media used for delivering IEC messages indicated that only MVU messages had a significant PE of 92 % in the eastern and fringe transmission patterns (95 % CI 42–99, p = 0.012, Additional file 7). In these areas, exposure to a radio message (in the previous 4 months), televised message, and leaflet or press article message were associated with non-significant PEs of 45, 48, and 79 %, respectively, and messages delivered on a poster or through another medium (*hiragasy*, puppets, or theatre) showed no PE. In the rest of the country, exposure to a radio, MVU, or television message was associated with non-significant PEs of 15, 22, and 26 %, respectively, and messages delivered on a leaflet/article or poster or through another medium produced no PE (Additional file 7).

## Discussion

These results show contrasting effectiveness among the four MCIs studied. LLINs provided a 41 % PE in areas where LLIN distribution campaigns occurred (except for the southern sites), which is consistent with the PE observed in efficacy studies [16, 17] and in a case–control study conducted concurrently in Madagascar [18]. The southern transmission pattern encompasses seven health districts and is characterized by semi-arid weather, a short rainy season, and cultural differences with the rest of the country. In this region, LLIN use was associated with an increased risk of infection, which may have been due to an indication bias related to the local LLIN distribution policy or cultural habits. Although the national strategy is supposed to be applied similarly in all regions, local initiatives could have occurred. For example, LLINs could have been directed to areas with a higher incidence of clinical malaria, or preferentially distributed to households of patients consulting for malaria. It is also possible that intrafamilial redistribution of LLINs to members most at risk of infection, e.g. feeble or sick individuals, have caused or contributed to the indication bias. It was not possible to know whether this bias hided an actual efficacy of LLINs in this areas, or if LLINs failed to protect users.

In the present study, a community PE for LLINs was not observed, which may be explained by a decrease of bioavailability of insecticides that are embedded in LLIN fibres, at least partly. This rapid decline of insecticides of LLINs in Madagascar has been observed previously [19]. A decrease, or a complete loss, of insecticides would indeed lead to a situation where the population of vectors has recovered his capacity to transmit the parasite to individuals not covered by a bed net while LLINs would keep an individual PE due to its barrier effect. The difference in PE between LLINs and NIBNs may be explained by the older age of NIBNs that go together with more physical damages, and/or by a residual insecticide effect—which would be nevertheless insufficient to translate into a community PE. It is also possible that the cut-off of 75 % that was used to divide LLIN coverage into high and low is not appropriate to demonstrate the community PE of LLINs. Nevertheless, previous studies showed that community protection offered by vector control interventions such as LLIN arises only when coverage reaches at least 50 %, and its value and significance increase with the coverage [20], suggesting that setting a high cut-off, such as 75 %, increases the probability to detect a community PE. Insect resistance to pyrethroids remains limited to a few foci in Madagascar and can hardly explain the absence of community PE for LLINs (Ratovonjato J. and Boyer S., personal communication, and [21]).

This study demonstrated a significant 44 % effectiveness against *Plasmodium* infection in households covered by IRS; however, this result was limited to the southern transmission pattern. This contrasts with the results from a contemporaneous case–control study that showed a 51 % PE in all transmission patterns [18]. The previous IRS campaign occurred approximately 1 year before the study, and this campaign would likely have had a limited or no impact on the prevalence of *Plasmodium* infections, except in the south where the duration of *Plasmodium* transmission is shorter. Thus, fewer intercurrent infections would have occurred since the previous IRS campaign [22, 23]. This result may also be related to the mechanism of IRS, which is more efficient at killing endophilic vectors (i.e., resting indoors after a blood meal) than at preventing people sleeping in a sprayed room from being bitten. Therefore, other authors have proposed that the protection offered by IRS is more visible at the community level than at the household level [24]. Indeed, these results showed an important and significant community-level PE of 78 % for high IRS coverage (≥75 %). This PE is similar to the PE observed in meta-analyses of efficacy studies against infection [17, 25]. The effectiveness of IRS could not be determined by comparing the PR between sprayed and non-sprayed areas in Madagascar because its deployment depends on the transmission level, i.e., the PR.

These results provide information on the effectiveness of distributing LLINs and performing IRS campaigns in combination. IRS only demonstrated a PE at the household level in the southern transmission pattern, i.e. precisely where the effectiveness of LLINs was not demonstrated. If LLINs were indeed not protective in the South, it is possible that IRS offset LLINs’ failure, which resulted in an increase of its PE. This enhanced PE of IRS might have been reinforced by a lower coverage of LLINs in the South (43.6 versus 65.1 % in the East and 60.5 in the West, [5]) This sort of interference have been suggested to explain the absence of additive effects in trials [26, 27] or observational studies [28–30] aimed at evaluating the efficacy or effectiveness of this combination of MCIs. In the present study, in other areas where both IRS and LLINs were deployed, the PE of high IRS coverage was slightly increased for individuals using LLINs. This suggests, on the contrary, that these MCIs could have additive effects, as demonstrated in other trials [31, 32] or observational studies [4, 33]. Unfortunately, the small number of RDT-positive habitants (n = 5) at the sites with high IRS coverage brought down the statistical power of the analysis of the potential synergistic effect of these two interventions. Therefore, the significance of the difference of prevalence between the group benefitting from the two MCIs and the groups covered by one MCI only was not presented. Moreover, the use of LLINs at sites with low IRS coverage produced a small and non-significant PE, suggesting again that both MCIs could interfere with each other and rarely demonstrate independently its effectiveness in areas where the two MCIs are deployed. Overall, these results suggest that the effectiveness of the two interventions are not independent and that the interventions interact in a complex manner [34]. Integrated vector control must be considered whenever the coverage or effectiveness of one single intervention is questionable. In Madagascar, policy makers have extended the target zones of IRS campaigns towards the southern transmission pattern [35] where LLINs lacked effectiveness, which is consistent with the findings presented here.

These results showed a non-significant PE of 21 and 24 % for high and very high IEC exposure, respectively, against *Plasmodium* infection in two transmission patterns. Only one medium (MVU) was found to be significantly associated with reduced parasite infection in these two areas, and its PE was surprisingly high (92 %). Because the objective of IEC messages is to increase adherence to preventive and curative interventions, these behaviours are expected to reduce the individual likelihood of infection [36]. The lack of significant PE for most of the IEC media may be related to the smaller expected impact of mass media relative to community mobilization [36, 37] but may also be due to a lack of statistical power for detecting such a small PE. Because MVU reaches zones accessible by car [5] where malaria transmission might be lower, the apparently high PE of MVU could be related to a non-controlled confounding factor.

The present study suggests an important (66 %) PE for IPTp against malaria infection, although the value was not significant because the analysis lacked power. IPTp is not designed to reduce the transmission of malaria but rather its burden in terms of maternal and neonatal morbidity and mortality. A contemporaneous case–control study conducted in Madagascar suggested PE of 73 % [18].

Certain interventions, such as LLINs, have been shown to offer significant protection to the young [38]; therefore, it was tested whether this factor could have affected their overall effectiveness. Results do not suggest that MCIs are more or less effective according to age. Significant interactions were found between transmission patterns and control interventions. However, these interactions did not reflect the partition of the country into low versus high transmission areas, which is inconsistent with meta-analyses of efficacy studies possibly because these meta-analyses focus on effectiveness against clinical outcomes and not infection [16, 39] or due to the study sites, which were smaller and more homogeneous in terms of malaria transmission.

This study provides data on the effectiveness of MCIs against *Plasmodium* infection in the context of malaria pre-elimination, which is part of the Malagasy health policy [1]. PE values close to the values measured in controlled efficacy trials indicate the preservation of MCI efficacy but do not guarantee their impact on malaria transmission and burden. The inappropriate delivery of preventive interventions in terms of coverage and quality may contribute to the resurgence of malaria [19].

A limitation of this study was the selection of study sites, which was not random but centred on SHCs. Nevertheless, the sample includes populations from all epidemiologic and sociocultural groups in Madagascar [5], and analytical epidemiology does not require the sample to be strictly representative [40]. The important variability in transmission patterns and seasons throughout the study may have interfered with the analysis, but the use of multilevel models hopefully overcomes this drawback. Another limitation is the use of RDTs for determining parasite prevalence, instead of more sensitive techniques like molecular biology. The probability for this limitation to have induced a bias in the evaluation of the PE of MCIs is low for several reasons. First, the validity of the RDT used in this study was demonstrated in Madagascar [41]. Then, since parasitaemia was a relatively rare event (<5 % in all transmission patterns [5]), the proportion of ‘true negative’ in the RDT negative population is likely considerable and virtually reclassifying the few ‘false negative’ individuals would not influence greatly the measure of the PE. Finally, RDT miss sensitivity especially for individuals with low parasitaemia [42]; this low parasitaemia may be related to a greater exposure to MCIs as compared with RDT positive individuals, thus false negative individuals stand between ‘true positive’ and ‘true negative’ both in terms of parasitaemia and MCI exposure. Therefore, the effect of the bias mentioned above might be even more attenuated.

This is the first comprehensive study measuring the post-deployment effectiveness of a complete portfolio of MCIs within even a single area let alone an entire country, and it is the first study with this level of representativeness to include all ages and genders. Other major advantages include the large sample size and adjustment for socio-economic factors. This survey design is critical for policy making and impact forecasting because modelling studies based on efficacy trials generally do not consider the complexity and heterogeneity of *Plasmodium* transmission [15].

## Conclusions

This study presents a unique countrywide and population-wide survey designed to provide a complete snapshot of the effectiveness of all MCIs in Madagascar during 2012–2013 with the objective of malaria pre-elimination. A PE value for LLINs close to the PE observed in controlled trials suggests that their efficacy is largely preserved in Madagascar, except in one transmission pattern. However, their community PE might be affected. If a rapid loss of insecticide activity in LLIN fibres is confirmed, policy makers should consider more frequent distribution campaigns. Additional IRS campaigns are also indicated where and when the effectiveness of LLINs is questionable. Overall, these results confirm the importance of integrated vector control for malaria control policies in Madagascar. Nevertheless, policy makers should adapt MCI strategies to social and epidemiological characteristics at a subnational level. In particular, an effectiveness analysis at the country scale and stratified for local transmission patterns should be conducted wherever MIS or similar data are available.

## Additional files

10.1186/s12936-016-1376-5 Definition of variables.
10.1186/s12936-016-1376-5 Multivariate models bed nets.
10.1186/s12936-016-1376-5 Multivariate models IRS.
10.1186/s12936-016-1376-5 Multivariate models IRS and LLIN.
10.1186/s12936-016-1376-5 Multivariate models IPTp.
10.1186/s12936-016-1376-5 Multivariate models IEC.
10.1186/s12936-016-1376-5 Models IEC by media.

## References

[1] Ministère de la Santé Publique. Plan Stratégique de Lutte contre le Paludisme 2007–2012—Du contrôle vers l’élimination du paludisme à Madagascar. Antananarivo; 2007.

[2] WHO Global Malaria Programme (2013). World Malaria Report 2013.

[3] Lim SS, Fullman N, Stokes A, Ravishankar N, Masiye F, Murray CJL (2011). Net benefits: a multicountry analysis of observational data examining associations between insecticide-treated mosquito nets and health outcomes. PLoS Med.

[4] Fullman N, Burstein R, Lim SS, Medlin C, Gakidou E (2013). Nets, spray or both? The effectiveness of insecticide-treated nets and indoor residual spraying in reducing malaria morbidity and child mortality in sub-Saharan Africa. Malar J.

[5] Kesteman T, Randrianarivelojosia M, Mattern C, Raboanary E, Pourette D, Girond F (2014). Nationwide evaluation of malaria infections, morbidity, mortality, and coverage of malaria control interventions in Madagascar. Malar J.

[6] Nankabirwa J, Brooker SJ, Clarke SE, Fernando D, Gitonga CW, Schellenberg D (2014). Malaria in school-age children in Africa: an increasingly important challenge. Trop Med Int Health.

[7] Randrianasolo L, Raoelina Y, Ratsitorahina M, Ravolomanana L, Andriamandimby S, Heraud J-M (2010). Sentinel surveillance system for early outbreak detection in Madagascar. BMC Public Health.

[8] Institut National de la Statistique, Programme National de Lutte contre le Paludisme, ICF International. Enquête sur les indicateurs du paludisme à Madagascar 2011. Antananarivo; 2012.

[9] Harrell FEJ. bpower—Power and sample size for two-sample binomial test. Packag Hmisc. 2014. p. 28–30.

[10] Filmer D, Pritchett LH (2001). Estimating wealth effects without expenditure data—or tears: an application to educational enrollments in states of India. Demography.

[11] Worldpop. http://www.worldpop.org.uk/. Accessed 13 June 2014. (Archived by WebCite^®^ at http://www.webcitation.org/6QIczygsd).

[12] Carey VJ, Lumley T, Ripley B. gee. Generalized Estimation Equation solver. R package version 4.13–18; 2012.

[13] Gelman A, Goegebeur Y, Tuerlinckx F, Van Mechelen I (2000). Diagnostic checks for discrete data regression models using posterior predictive simulations. Appl Stat.

[14] Gelman A, Su Y-S. Arm: Data Analysis Using Regression and Multilevel/Hierarchical Models. R package version 1.6–09; 2013.

[15] Lengeler C, Snow R (1996). From efficacy to effectiveness: insecticide-treated bednets in Africa. Bull World Health Organ.

[16] Lengeler C (2004). Insecticide-treated bed nets and curtains for preventing malaria. Cochrane Database Syst Rev.

[17] Eisele TP, Larsen D, Steketee RW (2010). Protective efficacy of interventions for preventing malaria mortality in children in *Plasmodium falciparum* endemic areas. Int J Epidemiol.

[18] Kesteman T, Randrianarivelojosia M, Raharimanga V, Randrianasolo L, Piola P, Rogier C (2016). Effectiveness of malaria control interventions in Madagascar: a nationwide case-control survey. Malar J.

[19] Kesteman T, Rafalimanantsoa SA, Razafimandimby H, Rasamimanana HH, Raharimanga V, Ramarosandratana B (2016). Multiple causes of an unexpected malaria outbreak in a high-transmission area in Madagascar. Malar J.

[20] Larsen DA, Hutchinson P, Bennett A, Yukich J, Anglewicz P, Keating J (2014). Community coverage with insecticide-treated mosquito nets and observed associations with all-cause child mortality and malaria parasite infections. Am J Trop Med Hyg.

[21] Ratovonjato J, Le Goff G, Rajaonarivelo E, Rakotondraibe EM, Robert V (2003). [Recent observations on the sensitivity to pyrethroids and DDT of *Anopheles arabiensis* and *Anopheles funestus* in the central Highlands of Madagascar; preliminary results on the absence of the kdr mutation in *An. arabiensis*] (in French). Arch Inst Pasteur Madag.

[22] Bukirwa H, Yau V, Kigozi R, Filler S, Quick L, Lugemwa M (2009). Assessing the impact of indoor residual spraying on malaria morbidity using a sentinel site surveillance system in Western Uganda. Am J Trop Med Hyg.

[23] Bradley J, Matias A, Schwabe C, Vargas D, Monti F, Nseng G (2012). Increased risks of malaria due to limited residual life of insecticide and outdoor biting versus protection by combined use of nets and indoor residual spraying on Bioko Island, Equatorial Guinea. Malar J.

[24] Rehman AM, Coleman M, Schwabe C, Baltazar G, Matias A, Gomes IR (2011). How much does malaria vector control quality matter: the epidemiological impact of holed nets and inadequate indoor residual spraying. PLoS ONE.

[25] Kim D, Fedak K, Kramer R (2012). Reduction of malaria prevalence by indoor residual spraying: a meta-regression analysis. Am J Trop Med Hyg.

[26] Corbel V, Akogbeto M, Damien GB, Djenontin A, Chandre F, Rogier C (2012). Combination of malaria vector control interventions in pyrethroid resistance area in Benin: a cluster randomised controlled trial. Lancet Infect Dis.

[27] Pinder M, Jawara M, Jarju LBS, Salami K, Jeffries D, Adiamoh M (2015). Efficacy of indoor residual spraying with dichlorodiphenyltrichloroethane against malaria in Gambian communities with high usage of long-lasting insecticidal mosquito nets: a cluster-randomised controlled trial. Lancet.

[28] Rehman AM, Mann AG, Schwabe C, Reddy MR, Roncon Gomes I, Slotman MA (2013). Five years of malaria control in the continental region, Equatorial Guinea. Malar J.

[29] Temu EA, Coleman M, Abilio AP, Kleinschmidt I (2012). High prevalence of malaria in Zambezia, Mozambique: the protective effect of IRS versus increased risks due to pig-keeping and house construction. PLoS ONE.

[30] Skarbinski J, Mwandama D, Wolkon A, Luka M, Jafali J, Smith A (2012). Impact of indoor residual spraying with lambda-cyhalothrin on malaria parasitemia and anemia prevalence among children less than five years of age in an area of intense, year-round transmission in Malawi. Am J Trop Med Hyg.

[31] Hamel MJ, Otieno P, Bayoh N, Kariuki S, Were V, Marwanga D (2011). The combination of indoor residual spraying and insecticide-treated nets provides added protection against malaria compared with insecticide-treated nets alone. Am J Trop Med Hyg.

[32] West PA, Protopopoff N, Wright A, Kivaju Z, Tigererwa R, Mosha FW (2014). Indoor residual spraying in combination with insecticide-treated nets compared to insecticide-treated nets alone for protection against malaria: a cluster randomised trial in Tanzania. PLoS Med.

[33] Kleinschmidt I, Schwabe C, Shiva M, Segura JL, Sima V, Mabunda SJA (2009). Combining indoor residual spraying and insecticide-treated net interventions. Am J Trop Med Hyg.

[34] Vector Control Technical Expert Group. WHO guidance for countries on combining indoor residual spraying and long-lasting insecticidal nets. Geneva; 2014.

[35] Ministère de la Santé Publique. Plan Stratégique de Lutte contre le Paludisme 2013–2017—Renforcer les acquis du contrôle en vue de l’élimination du paludisme à Madagascar. Antananarivo; 2012.

[36] Ministère de la Santé Publique, Programme National de Lutte contre le Paludisme. Revue du Programme Paludisme à Madagascar. Antananarivo; 2011.

[37] Mattern C, Pourette D, Raboanary E, Kesteman T, Piola P, Randrianarivelojosia M (2016). “Tazomoka is not a problem”. Local perspectives on malaria, fever case management and bed net use in Madagascar. PLoS ONE.

[38] Satoguina J, Walther B, Drakeley C, Nwakanma D, Oriero EC, Correa S (2009). Comparison of surveillance methods applied to a situation of low malaria prevalence at rural sites in The Gambia and Guinea Bissau. Malar J.

[39] Pluess B, Tanser FC, Lengeler C, Sharp BL (2010). Indoor residual spraying for preventing malaria. Cochrane Database Syst Rev.

[40] Kleinbaum DG, Morgenstern H, Kupper LL (1981). Selection bias in epidemiologic studies. Am J Epidemiol.

[41] Ratsimbasoa A, Randriamanantena A, Raherinjafy R, Rasoarilalao N, Ménard D (2007). Which Malaria rapid test for Madagascar? Field And laboratory evaluation of three tests and expert microscopy of samples from suspected malaria patients in Madagascar. Am J Trop Med Hyg.

[42] Schachterle SE, Mtove G, Levens JP, Clemens EG, Shi L, Raj A (2011). Prevalence and density-related concordance of three diagnostic tests for malaria in a region of Tanzania with hypoendemic malaria. J Clin Microbiol.

