# Supplementary material for: Post-deployment effectiveness of malaria control interventions on Plasmodium infections in Madagascar: a comprehensive phase IV assessment
Source: Malar J. 2016 Jun 16;15:322. doi: 10.1186/s12936-016-1376-5 (PMC4910239; doi:10.1186/s12936-016-1376-5)
Supplement: Supplementary file 1 — 10.1186/s12936-016-1376-5 Definition of variables. [file 12936_2016_1376_MOESM1_ESM.docx]

**Definition of variables**

The head of household or a representative answered a questionnaire, which included all household members' age and sex, housing features, living conditions, asset ownership, previous IRS details, and bed net ownership, including the characteristics (LLIN or non-impregnated bed net [NIBN]) and use. All of the participants were asked to answer a questionnaire about socio-demographic features and bed net usage over the preceding 3 months. Individuals ≥15 years old were asked about their exposure to IEC messages pertaining to malaria (media type and time since last event) and management of current or prior pregnancies (antenatal care, IPTp), if applicable.

We estimated the individual coverage of LLINs using two methods: (i) whether the individual was listed among household members that had slept under a LLIN the previous night according to the head of the household or his representative (household questionnaire) and (ii) according to the answer to the question “How many nights did you spend under a mosquito net during the last 3 months?” In the latter question, the answer “every night” was inconsistent with any other answer (“1-6 time(s)/week”, “1-4 time(s)/month”, “less than once a month or never”). We defined individuals as “every night LLIN users” if they answered “every night” to the question above and were listed among LLIN users in the household questionnaire. Bed net users for whom the characteristics of the bed net were not known, who answered “every night” to the question above and were not listed among bed net users in the household questionnaire were classified as non-impregnated bed net users.

We defined the coverage of IRS if the head of the household or his representative provided an affirmative answer to the question “Did someone visit your household in the last 12 months to spray walls to fight against malaria?” and reported a delay since the previous IRS campaign ≤20 months to include only spraying during the prior campaign. For visitors, the IRS status at home was considered. Because the IRS campaign was late in the 2012-2013 rainy season, none of the households had been sprayed in the study sites at time of the survey, and no subset of the households recently sprayed could be defined. To accurately determine the households covered by IRS, local designations (e.g., “DDT”) were used instead of or in addition to the term “spraying walls to fight against malaria”.

We considered a woman to have been covered by IPTp if she reported having taken at least two doses of SP during the course of antenatal care in a ≥4-month ongoing pregnancy or during her previous pregnancy if she delivered within the last 12 months. Males and females not of childbearing age (15-50 years old), nulliparous women, women pregnant for <4 months, and women who had delivered more than 12 months prior were excluded from the calculation of IPTp effectiveness.

We considered the following media for the evaluation of exposure to IEC messages about malaria: radio (≤1 month, >1 to ≤4 months, or >4 months since the last message heard about malaria), poster (≤1 month, >1 to ≤4 months, or >4 months since the last poster seen), mobile video unit (≤1 year or >1 year since the last time seen), television (≤4 months or >4 months since the last message seen), leaflet or written press article (≤1 year or >1 year since the last message read), and other media/presentation and the time since the last event (e.g., *hiragasy* [traditional Malagasy theatre], puppets, and theatre). For 50 individuals, exposure to IEC media could not be determined.

Household socio-economic status (SES) was calculated using the first principal component score based on variables related to house size, living conditions (toilets, waste, water, lighting, and cooking fuel), 13-asset ownership, and household utilities. The scores were converted to wealth quintiles for analysis. An index of permeability of housing to mosquitoes was calculated using the first principal component score based on variables related to housing construction materials (walls, roof, and floor) and the presence and size of holes in the housing structure. The scores were converted to permeability quintiles for analysis.

The level of education was not provided for 23 persons above the age of 15, and they were categorized as people with no formal education. For children under 15 years, the education level and the IEC exposure level of the caretaker were assigned.
