# Supplementary material for: Post-deployment effectiveness of malaria control interventions on Plasmodium infections in Madagascar: a comprehensive phase IV assessment
Source: Malar J. 2016 Jun 16;15:322. doi: 10.1186/s12936-016-1376-5 (PMC4910239; doi:10.1186/s12936-016-1376-5)
Supplement: Supplementary file 2 — 10.1186/s12936-016-1376-5 Multivariate models bed nets. [file 12936_2016_1376_MOESM2_ESM.docx]

## Complete multivariate models for bed nets

|  | **Other transmission patterns** | | | | |  | **Southern transmission pattern** | | | | |
| --- | --- | --- | --- | --- | --- | --- | --- | --- | --- | --- | --- |
| **Variable** | **Category** | **N** | **% RDT+** | **Adj. OR [95% CI]** | **p** |  | **Category** | **N** | **% RDT+** | **Adj. OR [95% CI]** | **p** |
| **Nightly bed net use** | LLIN | 6780 | 3·7 | 0·59 [0·46-0·77] | <0·001 |  | LLIN | 397 | 5·5 | 4·45 [2·10-9·40] | <0·001 |
|  | NIBN | 1364 | 3·7 | 0·76 [0·55-1·05] | 0·09 |  | NIBN | 44 | 2·3 | 3·09 [0·11-88·41] | 0·509 |
|  | None | 4391 | 4·4 | 1·00 |  |  | None | 516 | 1·2 | 1·00 |  |
| **LLIN coverage** | ≤75% | 11327 | 3·7 | 1·00 |  |  | ≤75% | 957 | 3·0 | NA |  |
|  | >75% | 1208 | 6·2 | 1·82 [0·51-6·49] | 0·358 |  | >75% | 0 | NA | NA |  |
| **Age group** | 0-1 year | 587 | 3·2 | 1·75 [0·83-3·70] | 0·141 |  | - | - | - | - |  |
|  | 2-4 years | 1373 | 3·8 | 2·31 [1·40-3·83] | 0·001 |  | 0-4 years | 207 | 1·4 | 0·57 [0·22-1·49] | 0·252 |
|  | 5-9 years | 2186 | 6·6 | 4·05 [2·74-5·97] | <0·001 |  | 5-9 years | 203 | 3·4 | 1·85 [0·82-4·17] | 0·136 |
|  | 10-14 years | 1767 | 6·3 | 4·03 [2·68-6·06] | <0·001 |  | 10-15 years | 172 | 7·0 | 3·76 [2·18-6·48] | <0·001 |
|  | 15-19 years | 1305 | 4·3 | 2·82 [1·85-4·31] | <0·001 |  | 15-19 years | 72 | 2·8 | 1·32 [0·30-5·81] | 0·711 |
|  | 20-39 years | 3049 | 2·5 | 1·57 [1·05-2·35] | 0·029 |  | 20-39 years | 173 | 0·6 | 0·24 [0·09-0·62] | 0·003 |
|  | ≥40 years | 2268 | 1·6 | 1·00 |  |  | ≥40 years | 130 | 3·1 | 1·00 |  |
| **Sex** | Male | 5436 | 5·0 | 1·00 |  |  | Male | 392 | 2·8 | 1·00 |  |
|  | Female | 7099 | 3·1 | 0·66 [0·54-0·81] | <0·001 |  | Female | 565 | 3·2 | 1·16 [0·76-1·77] | 0·492 |
| **Education level** | None or unknown | 1959 | 5·6 | 1·86 [1·15-3·01] | 0·012 |  | None or unknown | 569 | 4·0 | 1·63 [0·44-6·04] | 0·464 |
|  | Primary | 5396 | 4·8 | 1·95 [1·25-3·05] | 0·003 |  | Primary | 188 | 2·1 | 1·08 [0·21-5·48] | 0·922 |
|  | Lower secondary | 3533 | 2·8 | 1·40 [0·89-2·21] | 0·147 |  | Secondary or above | 200 | 1·0 | 1·00 |  |
|  | Upper secondary/tertiary | 1647 | 1·7 | 1·00 |  |  | - | - | - | - |  |
| **SES quintile** | 1^st^ (poorest) | 2480 | 7·0 | 2·71 [1·50-4·88] | <0·001 |  | 1^st^ (poorest) | 484 | 3·3 | 0·43 [0·08-2·35] | 0·332 |
|  | 2^nd^ | 2655 | 5·1 | 2·38 [1·43-3·98] | <0·001 |  | 2^nd^ | 206 | 5·3 | 1·17 [0·20-6·88] | 0·862 |
|  | 3^rd^ | 2430 | 3·2 | 1·62 [0·97-2·71] | 0·064 |  | 3^rd^ | 155 | 0·6 | 0·72 [0·35-1·48] | 0·368 |
|  | 4^th^ | 2441 | 2·5 | 1·62 [1·09-2·40] | 0·016 |  | 4^th^ & 5^th^ (wealthiest) | 112 | 0·9 | 1·00 |  |
|  | 5^th^ (wealthiest) | 2529 | 1·8 | 1·00 |  |  | - | - | - | - |  |
| **Population density** | Low (rural) | 8200 | 4·6 | 1·16 [0·40-3·38] | 0·78 |  | Low (rural) | 489 | 5·3 | 11·47 [2·25-58·47] | 0·003 |
|  | Medium | 3401 | 2·4 | 1·06 [0·38-2·99] | 0·91 |  | Medium | 468 | 0·6 | 1·00 |  |
|  | High (urban) | 934 | 3·4 | 1·00 |  |  | High (urban) | 0 | NA | NA |  |
| **Transmission pattern** | East | 3451 | 4·6 | 13·99 [3·16-61·89] | <0·001 |  | - | - | - | - |  |
|  | Fringe | 2430 | 0·7 | 1·00 |  |  | - | - | - | - |  |
|  | West | 6654 | 4·7 | 12·76 [2·93-55·58] | <0·001 |  | - | - | - | - |  |

Association between RDT positivity and bed net use or bed net coverage in multivariate analyses in the southern transmission pattern or in zones targeted for LLIN distribution excluding the southern transmission pattern. NA: not applicable.
