# Supplementary material for: Post-deployment effectiveness of malaria control interventions on Plasmodium infections in Madagascar: a comprehensive phase IV assessment
Source: Malar J. 2016 Jun 16;15:322. doi: 10.1186/s12936-016-1376-5 (PMC4910239; doi:10.1186/s12936-016-1376-5)
Supplement: Supplementary file 3 — 10.1186/s12936-016-1376-5 Multivariate models IRS. [file 12936_2016_1376_MOESM3_ESM.docx]

## Complete multivariate models for IRS

|  | **Other transmission patterns** | | | | |  | **Southern transmission pattern** | | | | |
| --- | --- | --- | --- | --- | --- | --- | --- | --- | --- | --- | --- |
| **Variable** | **Category** | **N** | **% RDT+** | **Adj. OR [95% CI]** | **p** |  | **Category** | **N** | **% RDT+** | **Adj. OR [95% CI]** | **p** |
| **IRS the previous year** | No | 1736 | 1·1 | 1·00 |  |  | No | 303 | 4·3 | 1·00 |  |
|  | Yes | 3460 | 1·4 | 1·11 [0·69-1·78] | 0·669 |  | Yes | 654 | 2·4 | 0·56 [0·35-0·89] | 0·014 |
| **IRS coverage** | ≤75% | 3662 | 1·6 | 1·00 |  |  | ≤75% | 708 | 3·7 | 1·00 |  |
|  | >75% | 1534 | 0·6 | 0·20 [0·07-0·56] | 0·002 |  | >75% | 249 | 1·2 | 0·14 [0·03-0·61] | 0·008 |
| **Age group** | 0-1 year | 212 | 2·4 | 3·07 [1·23-7·64] | 0·016 |  | - | - | - | - |  |
|  | 2-4 years | 585 | 0·7 | 0·77 [0·14-4·10] | 0·758 |  | 0-4 years | 207 | 1·4 | 0·58 [0·23-1·51] | 0·266 |
|  | 5-9 years | 905 | 2·0 | 2·32 [0·83-6·43] | 0·107 |  | 5-9 years | 203 | 3·4 | 1·56 [0·62-3·93] | 0·343 |
|  | 10-14 years | 774 | 1·4 | 1·68 [0·74-3·82] | 0·214 |  | 10-15 years | 172 | 7·0 | 3·11 [1·40-6·91] | 0·005 |
|  | 15-19 years | 521 | 1·3 | 1·85 [0·69-4·96] | 0·224 |  | 15-19 years | 72 | 2·8 | 1·18 [0·30-4·60] | 0·814 |
|  | 20-39 years | 1206 | 1·2 | 1·49 [0·70-3·14] | 0·3 |  | 20-39 years | 173 | 0·6 | 0·25 [0·09-0·67] | 0·006 |
|  | ≥40 years | 993 | 0·8 | 1·00 |  |  | ≥40 years | 130 | 3·1 | 1·00 |  |
| **Sex** | Male | 2331 | 1·8 | 1·00 |  |  | Male | 392 | 2·8 | 1·00 |  |
|  | Female | 2865 | 0·9 | 0·48 [0·29-0·79] | 0·004 |  | Female | 565 | 3·2 | 1·12 [0·85-1·49] | 0·423 |
| **Education level** | None or unknown | 458 | 1·7 | 7·16 [1·43-36·01] | 0·017 |  | None or unknown | 569 | 4·0 | 1·97 [0·61-6·36] | 0·258 |
|  | Primary | 2763 | 1·3 | 5·95 [1·17-30·33] | 0·032 |  | Primary | 188 | 2·1 | 1·56 [0·32-7·71] | 0·584 |
|  | Lower secondary | 1389 | 1·5 | 5·26 [1·23-22·60] | 0·026 |  | Secondary or above | 200 | 1·0 | 1·00 |  |
|  | Upper secondary/tertiary | 586 | 0·3 | 1·00 |  |  | - | - | - | - |  |
| **SES quintile** | 1^st^ (poorest) | 990 | 1·8 | 0·94 [0·43-2·05] | 0·878 |  | 1^st^ (poorest) | 484 | 3·3 | 1·15 [0·67-1·97] | 0·62 |
|  | 2^nd^ | 1145 | 0·9 | 0·60 [0·22-1·64] | 0·318 |  | 2^nd^ | 206 | 5·3 | 2·06 [1·59-2·66] | <0·001 |
|  | 3^rd^ | 1333 | 1·0 | 0·82 [0·43-1·53] | 0·527 |  | 3^rd^ | 155 | 0·6 | 0·78 [0·41-1·50] | 0·46 |
|  | 4^th^ | 1216 | 1·7 | 1·36 [0·82-2·25] | 0·227 |  | 4^th^ & 5^th^ (wealthiest) | 112 | 0·9 | 1·00 |  |
|  | 5^th^ (wealthiest) | 512 | 1·0 | 1·00 |  |  | - | - | - | - |  |
| **Population density** | Low (rural) | 4033 | 1·2 | 1·28 [0·90-1·84] | 0·173 |  | Low (rural) | 489 | 5·3 | - * |  |
|  | Medium | 1066 | 1·5 | 1·71 [0·39-7·56] | 0·476 |  | Medium | 468 | 0·6 | - * |  |
|  | High (urban) | 97 | 3·1 | 1·00 |  |  | High (urban) | 0 | NA | - |  |
| **Transmission pattern** | Fringe | 2430 | 0·7 | 1·00 |  |  | - | - | - | - |  |
|  | Highlands | 1259 | 1·7 | 2·89 [0·98-8·49] | 0·053 |  | - | - | - | - |  |
|  | West | 1507 | 1·9 | 4·44 [1·34-14·78] | 0·015 |  | - | - | - | - |  |

Association between RDT positivity and IRS in the household or IRS coverage in multivariate analyses in the southern transmission pattern or in zones targeted by IRS campaigns excluding the southern transmission pattern. * The population density and IRS coverage could not be included in the model because of collinearity. NA: not applicable.
