# Supplementary material for: Post-deployment effectiveness of malaria control interventions on Plasmodium infections in Madagascar: a comprehensive phase IV assessment
Source: Malar J. 2016 Jun 16;15:322. doi: 10.1186/s12936-016-1376-5 (PMC4910239; doi:10.1186/s12936-016-1376-5)
Supplement: Supplementary file 4 — 10.1186/s12936-016-1376-5 Multivariate models IRS and LLIN. [file 12936_2016_1376_MOESM4_ESM.docx]

## Complete models for IRS and LLIN, excluding the south

|  |  |  | **% RDT+** | **Bivariate** | |  | **Multivariate** | | |
| --- | --- | --- | --- | --- | --- | --- | --- | --- | --- |
| **Variable** | **Category** | **N** |  | **Crude OR [95% CI]** | **p** |  | **Adj. OR [95% CI]** | | **p** |
| **Nightly bed net use and/or IRS coverage the previous year** | LLIN use and IRS coverage >75% | 427 | 0·5 | 0·22 [0·04-1·23] | 0·085 |  | | 0·14 [0·03-0·61] | 0·009 |
|  | No LLIN use and IRS coverage >75% | 596 | 0·5 | 0·42 [0·10-1·82] | 0·248 |  | | 0·24 [0·07-0·83] | 0·024 |
|  | LLIN use and IRS coverage ≤75% | 1104 | 1·3 | 0·78 [0·44-1·37] | 0·385 |  | | 0·93 [0·58-1·49] | 0·764 |
|  | NIBN use and IRS coverage ≤75% | 160 | 1·9 | 0·96 [0·08-11·71] | 0·974 |  | | 0·98 [0·09-10·67] | 0·985 |
|  | No bed net use and IRS coverage ≤75% | 1650 | 1·4 | 1·00 |  |  | | 1·00 |  |
| **Age group** | 0-1 year | 169 | 1·8 | 2·84 [1·19-6·80] | 0·019 |  | | 2·60 [0·93-7·26] | 0·069 |
|  | 2-4 years | 454 | 0·7 | 0·95 [0·09-9·65] | 0·966 |  | | 0·87 [0·10-7·31] | 0·899 |
|  | 5-9 years | 671 | 2·1 | 3·08 [0·72-13·23] | 0·131 |  | | 2·89 [0·74-11·40] | 0·128 |
|  | 10-14 years | 562 | 1·8 | 2·55 [0·92-7·05] | 0·071 |  | | 2·33 [0·88-6·19] | 0·089 |
|  | 15-19 years | 412 | 1·2 | 1·75 [0·39-7·75] | 0·464 |  | | 1·92 [0·53-6·93] | 0·318 |
|  | 20-39 years | 933 | 0·5 | 0·76 [0·68-0·84] | <0·001 |  | | 0·78 [0·64-0·96] | 0·019 |
|  | ≥40 years | 736 | 0·7 | 1·00 |  |  | | 1·00 |  |
| **Sex** | Male | 1775 | 1·7 | 1·00 |  |  | | 1·00 |  |
|  | Female | 2162 | 0·7 | 0·41 [0·23-0·73] | 0·002 |  | | 0·45 [0·26-0·78] | 0·005 |
| **Education level** | None or unknown | 380 | 1·8 | 7·48 [2·06-27·06] | 0·002 |  | | 8·38 [1·44-48·69] | 0·018 |
|  | Primary | 2046 | 1·3 | 5·95 [1·87-19·00] | 0·003 |  | | 6·56 [1·32-32·56] | 0·021 |
|  | Lower secondary | 1054 | 1·0 | 4·55 [2·08-9·94] | <0·001 |  | | 4·84 [1·75-13·44] | 0·002 |
|  | Upper secondary/tertiary | 457 | 0·2 | 1·00 |  |  | | 1·00 |  |
| **SES quintile** | 1^st^ (poorest) | 861 | 1·7 | 2·63 [1·02-6·81] | 0·046 |  | | 0·92 [0·31-2·70] | 0·875 |
|  | 2^nd^ | 905 | 1·0 | 1·61 [0·50-5·24] | 0·427 |  | | 0·66 [0·18-2·48] | 0·54 |
|  | 3^rd^ | 832 | 0·8 | 1·75 [0·65-4·67] | 0·265 |  | | 0·90 [0·34-2·35] | 0·822 |
|  | 4^th^ | 916 | 1·2 | 1·88 [0·70-5·03] | 0·211 |  | | 1·23 [0·55-2·76] | 0·612 |
|  | 5^th^ (wealthiest) | 423 | 0·7 | 1·00 |  |  | | 1·00 |  |
| **Population density** | Low (rural) | 3220 | 1·2 | 0·97 [0·25-3·67] | 0·961 |  | | 1·31 [0·87-1·96] | 0·193 |
|  | Medium | 620 | 0·5 | 0·67 [0·18-2·52] | 0·559 |  | | 1·33 [0·42-4·27] | 0·63 |
|  | High (urban) | 97 | 3·1 | 1·00 |  |  | | 1·00 |  |
| **Transmission pattern** | Fringe | 2430 | 0·7 | 1·00 |  |  | | 1·00 |  |
|  | West | 1507 | 1·9 | 2·77 [0·75-10·31] | 0·128 |  | | 4·58 [1·69-12·39] | 0·003 |

Bivariate and multivariate analyses of factors associated with RDT positivity (RDT+) including IRS coverage and/or LLIN use in zones targeted by both IRS and LLIN campaigns but excluding the southern transmission pattern.
