# Supplementary material for: Post-deployment effectiveness of malaria control interventions on Plasmodium infections in Madagascar: a comprehensive phase IV assessment
Source: Malar J. 2016 Jun 16;15:322. doi: 10.1186/s12936-016-1376-5 (PMC4910239; doi:10.1186/s12936-016-1376-5)
Supplement: Supplementary file 5 — 10.1186/s12936-016-1376-5 Multivariate models IPTp. [file 12936_2016_1376_MOESM5_ESM.docx]

## Complete models for IPTp

|  |  |  | **% RDT+** | **Bivariate** | |  | **Multivariate** | |
| --- | --- | --- | --- | --- | --- | --- | --- | --- |
| **Variable** | **Category** | **N** |  | **Crude OR [95% CI]** | **p** |  | **Adj. OR [95% CI]** | **p** |
| **≥1 dose IPTp** | Yes | 103 | 1·9 | 0·48 [0·09-2·42] | 0·37 |  | 0·34 [0·05-2·10] | 0·244 |
|  | No | 104 | 3·8 | 1·00 |  |  | 1·00 |  |
| **Age group** | 15-24 years | 102 | 2·9 | 0·43 [0·07-2·81] | 0·378 |  | 0·33 [0·04-2·70] | 0·300 |
|  | 25-34 years | 79 | 1·3 | 0·17 [0·01-2·03] | 0·162 |  | 0·12 [0·01-1·55] | 0·105 |
|  | 35-49 years | 26 | 7·7 | 1·00 |  |  | 1·00 |  |
| **Parity** | Primiparous | 54 | 1·9 | 0·56 [0·06-4·89] | 0·599 |  | 0·66 [0·05-8·49] | 0·748 |
|  | Multiparous | 153 | 3·3 | 1·00 |  |  | 1·00 |  |
| **Education level** | None or unknown | 47 | 4·3 | 3·27 [0·29-36·93] | 0·338 |  | 1·47 [0·11-20·55] | 0·773 |
|  | Primary | 91 | 3·3 | 2·60 [0·29-23·68] | 0·395 |  | 1·65 [0·15-18·39] | 0·682 |
|  | Secondary or above | 69 | 1·4 | 1·00 |  |  | 1·00 |  |
| **SES** | Low (≤median) | 120 | 4·2 | 4·38 [0·62-30·75] | 0·137 |  | 3·06 [0·31-30·23] | 0·338 |
|  | High (>median) | 87 | 1·1 | 1·00 |  |  | 1·00 |  |

Bivariate and multivariate analyses of factors associated with RDT positivity (RDT+), including IPTp in zones where IPTp is recommended. Population density and transmission patterns could not be included because they caused a lack of convergence in the model.
