# Supplementary material for: Post-deployment effectiveness of malaria control interventions on Plasmodium infections in Madagascar: a comprehensive phase IV assessment
Source: Malar J. 2016 Jun 16;15:322. doi: 10.1186/s12936-016-1376-5 (PMC4910239; doi:10.1186/s12936-016-1376-5)
Supplement: Supplementary file 6 — 10.1186/s12936-016-1376-5 Multivariate models IEC. [file 12936_2016_1376_MOESM6_ESM.docx]

## Complete multivariate models for IEC

|  | **Other transmission patterns** | | | | |  | **Fringe and eastern transmission patterns** | | | | |
| --- | --- | --- | --- | --- | --- | --- | --- | --- | --- | --- | --- |
| **Variable** | **Category** | **N** | **% RDT+** | **Adj. OR [95% CI]** | **p** |  | **Category** | **N** | **% RDT+** | **Adj. OR [95% CI]** | **p** |
| **Exposure to malaria IEC messages** | Low | 4623 | 3·8 | 1·00 |  |  | Low | 2682 | 4·0 | 1·00 |  |
|  | High | 2497 | 4·0 | 1·08 [0·85-1·36] | 0·543 |  | High | 1779 | 2·6 | 0·79 [0·55-1·16] | 0·230 |
|  | Very high | 2432 | 3·5 | 0·98 [0·67-1·44] | 0·916 |  | Very high | 1413 | 1·7 | 0·76 [0·43-1·34] | 0·345 |
| **Age group** | 0-1 year | 431 | 2·8 | 1·62 [0·80-3·27] | 0·177 |  | 0-1 year | 278 | 2·9 | 1·44 [0·36-5·75] | 0·603 |
|  | 2-4 years | 1078 | 3·2 | 1·99 [1·19-3·32] | 0·009 |  | 2-4 years | 633 | 3·3 | 2·00 [0·87-4·63] | 0·104 |
|  | 5-9 years | 1692 | 5·9 | 3·58 [2·53-5·05] | <0·001 |  | 5-9 years | 1011 | 4·9 | 3·26 [1·68-6·32] | <0·001 |
|  | 10-14 years | 1413 | 6·1 | 3·66 [2·43-5·52] | <0·001 |  | 10-15 years | 808 | 4·5 | 3·40 [2·18-5·30] | <0·001 |
|  | 15-19 years | 972 | 4·0 | 2·30 [1·58-3·35] | <0·001 |  | 15-19 years | 593 | 3·5 | 2·95 [1·44-6·05] | 0·003 |
|  | 20-39 years | 2215 | 2·7 | 1·56 [1·14-2·14] | 0·006 |  | 20-39 years | 1482 | 1·9 | 1·34 [0·62-2·88] | 0·462 |
|  | ≥40 years | 1751 | 1·8 | 1·00 |  |  | ≥40 years | 1069 | 1·2 | 1·00 |  |
| **Sex** | Male | 4122 | 4·7 | 1·00 |  |  | Male | 2527 | 3·7 | 1·00 |  |
|  | Female | 5430 | 3·1 | 0·66 [0·53-0·82] | <0·001 |  | Female | 3347 | 2·5 | 0·66 [0·50-0·88] | 0·004 |
| **Education level** | None or unknown | 1976 | 5·3 | 1·73 [1·12-2·69] | 0·014 |  | None or unknown | 629 | 4·5 | 2·16 [0·89-5·21] | 0·088 |
|  | Primary | 3817 | 4·2 | 1·75 [1·14-2·67] | 0·010 |  | Primary | 2656 | 3·9 | 2·42 [1·12-5·22] | 0·024 |
|  | Lower secondary | 2470 | 3·0 | 1·45 [0·92-2·29] | 0·108 |  | Lower secondary | 1826 | 2·1 | 1·51 [0·89-2·57] | 0·125 |
|  | Upper secondary/tertiary | 1289 | 2·0 | 1·00 |  |  | Upper secondary/tertiary | 763 | 0·9 | 1·00 |  |
| **SES quintile** | 1^st^ (poorest) | 1988 | 6·4 | 2·36 [1·48-3·76] | <0·001 |  | 1^st^ (poorest) | 1077 | 5·6 | 2·94 [1·40-6·21] | 0·005 |
|  | 2^nd^ | 1999 | 4·7 | 2·07 [1·42-3·01] | <0·001 |  | 2^nd^ | 1092 | 4·3 | 2·42 [1·23-4·77] | 0·011 |
|  | 3^rd^ | 1920 | 3·1 | 1·56 [1·15-2·11] | 0·005 |  | 3^rd^ | 1214 | 2·0 | 1·29 [0·61-2·71] | 0·510 |
|  | 4^th^ | 1584 | 2·8 | 1·75 [1·42-2·15] | <0·001 |  | 4^th^ | 1456 | 2·1 | 1·50 [0·80-2·80] | 0·207 |
|  | 5^th^ (wealthiest) | 2061 | 1·8 | 1·00 |  |  | 5^th^ (wealthiest) | 1035 | 1·4 | 1·00 |  |
| **Population density** | Low (rural) | 5904 | 4·8 | 2·52 [1·10-5·73] | 0·028 |  | Low (rural) | 3796 | 3·4 | 0·55 [0·23-1·31] | 0·178 |
|  | Medium | 2899 | 2·6 | 2·43 [0·91-6·52] | 0·078 |  | Medium | 1657 | 1·4 | 0·52 [0·26-1·01] | 0·055 |
|  | High (urban) | 749 | 0·8 | 1·00 |  |  | High (urban) | 421 | 6·2 | 1·00 |  |
| **Transmission pattern** | Highlands | 1984 | 1·5 | 1·00 |  |  | East | 3449 | 4·6 | 9·73 [3·13-30·23] | <0·001 |
|  | South | 944 | 3·1 | 2·10 [0·45-9·70] | 0·342 |  | Fringe | 2425 | 0·7 | 1·00 |  |
|  | West | 6624 | 4·6 | 3·11 [1·33-7·24] | 0·009 |  | - | - | - | - |  |

Association between RDT positivity and the level of exposure to IEC messages about malaria in multivariate analyses in the fringe and eastern transmission patterns or in the rest of the country.
