# Supplementary material for: Post-deployment effectiveness of malaria control interventions on Plasmodium infections in Madagascar: a comprehensive phase IV assessment
Source: Malar J. 2016 Jun 16;15:322. doi: 10.1186/s12936-016-1376-5 (PMC4910239; doi:10.1186/s12936-016-1376-5)
Supplement: Supplementary file 7 — 10.1186/s12936-016-1376-5 Models IEC by media. [file 12936_2016_1376_MOESM7_ESM.docx]

## Partial multivariate models for IEC, by media

| **Exposure to IEC messages through** | **Other transmission patterns** | | | | |  | **Fringe and eastern transmission patterns** | | | |  |
| --- | --- | --- | --- | --- | --- | --- | --- | --- | --- | --- | --- |
|  | **Category** | **N** | **% RDT+** | **Adj. OR [95% CI]** | **p** |  | **N** | **% RDT+** | **Adj. OR [95% CI]** | **p** | |
| **Radio** | Previous 4 months | 334 | 3·6 | 0·85 [0·34-2·15] | 0·733 |  | 234 | 1·7 | 0·55 [0·19-1·58] | 0·270 | |
|  | >4 months ago | 106 | 0·9 | 0·34 [0·07-1·55] | 0·163 |  | 2124 | 2·3 | 1·01 [0·68-1·49] | 0·973 | |
|  | Never | 3020 | 3·9 | 1·00 |  |  | 3516 | 3·5 | 1·00 |  | |
| **Poster** | Previous 4 months | 234 | 3·4 | 0·99 [0·38-2·61] | 0·985 |  | 136 | 2·9 | 1·09 [0·50-2·39] | 0·831 | |
|  | >4 months ago | 1743 | 3·6 | 1·00 [0·64-1·57] | 0·983 |  | 963 | 1·6 | 0·83 [0·56-1·25] | 0·382 | |
|  | Never | 7575 | 3·9 | 1·00 |  |  | 4775 | 3·3 | 1·00 |  | |
| **Mobile Video Unit** | Ever | 202 | 3·0 | 0·78 [0·30-2·04] | 0·615 |  | 147 | 0·7 | 0·08 [0·01-0·58] | 0·012 | |
|  | Never | 9350 | 3·8 | 1·00 |  |  | 5727 | 3·1 | 1·00 |  | |
| **Television** | Ever | 1159 | 1·8 | 0·74 [0·38-1·42] | 0·365 |  | 571 | 0·9 | 0·52 [0·25-1·08] | 0·078 | |
|  | Never | 8393 | 4·1 | 1·00 |  |  | 5303 | 3·2 | 1·00 |  | |
| **Leaflet or press article*** | Ever | 230 | 3·5 | 1·09 [0·52-2·29] | 0·814 |  | 227 | 0·4 | 0·21 [0·03-1·48] | 0·117 | |
|  | Never | 7338 | 4·4 | 1·00 |  |  | 7631 | 2·7 | 1·00 |  | |
| **Other media/exhibition** | Ever | 82 | 3·7 | 1·30 [0·36-4·74] | 0·691 |  | 92 | 2·2 | 1·69 [0·45-6·34] | 0·439 | |
|  | Never | 9470 | 3·8 | 1·00 |  |  | 5782 | 3·0 | 1·00 |  | |

Association between RDT positivity and exposure to IEC messages on malaria by different media in multivariate analyses in the fringe and eastern transmission patterns or in the rest of the country. OR were adjusted according to age, gender, education, SES quintile, population density, and transmission pattern. * Separate analyses were conducted for the highlands, fringe and eastern transmission patterns, and other transmission patterns.
